# Interictal Abnormalities of Neuromagnetic Gamma Oscillations in Migraine Following Negative Emotional Stimulation

**DOI:** 10.3389/fnbeh.2018.00169

**Published:** 2018-08-17

**Authors:** Ting Wu, Jie Fan, Yueqiu Chen, Jing Xiang, Donglin Zhu, Junpeng Zhang, Jingping Shi, Tianzi Jiang

**Affiliations:** ^1^Department of Neurology, Nanjing Brain Hospital, Nanjing Medical University, Nanjing, China; ^2^Department of Neurology, Cincinnati Children’s Hospital Medical Center, Cincinnati, OH, United States; ^3^Department of Medical Information and Engineering, School of Electrical Engineering and Information, Sichuan University, Chengdu, China; ^4^Department of Neurology, School of Medicine, Nanjing University, Nanjing, China; ^5^Key Laboratory for NeuroInformation of the Ministry of Education, School of Life Sciences and Technology, University of Electronic Science and Technology of China, Chengdu, China

**Keywords:** migraine, magnetoencephalography, emotional stimulation, gamma oscillation, M100, M170

## Abstract

Here, we aimed to investigate brain activity in migraineurs in response to emotional stimulation. Magnetoencephalography (MEG) was used to examine 20 patients with episodic migraine (EM group), 15 patients with chronic migraine (CM group), and 35 healthy participants (control group). Neuromagnetic brain activity was elicited by emotional stimulation using photographs of facial expressions. We analyzed the latency and amplitude of M100 and M170 components and used Morlet wavelet and beamformers to analyze the spectral and spatial signatures of MEG signals in gamma band (30–100 Hz). We found that the timing and frequency of MEG activity differed across the three groups in response negative emotional stimuli. First, peak M170 amplitude was significantly lower in the CM group than in the control group. Second, compared with the control group, the average spectral power was significantly lower in the EM group and CM group at M100 and M170. Third, the average spectral powers of the M100 and M170 in the CM group were negatively correlated with either HAM-D scores or migraine attack frequency. No significant differences across groups was found for positive or neutral emotional stimuli. Furthermore, after negative emotional stimuli, the MEG source analysis demonstrated that the CM group showed a significantly higher percentage of amygdala activation than the control group for M100 and M170. Thus, during headache free phases, migraineurs have abnormal brain activity in the gamma band in response to negative emotional stimuli.

**Trial Registration:** ChiCTR-RNC-17012599. Registered 7 September, 2017.

## Introduction

Migraine is a common and disabling headache disorder that can be episodic [episodic migraine (EM)] or chronic [chronic migraine (CM)] (Buse et al., 2013; Headache Classification Committee of the International Headache, 2013). Patients with CM have headaches at least 15 days a month, with at least 8 days a month in which their headaches and associated symptoms meet diagnostic criteria for migraine (Headache Classification Committee of the International Headache, 2013). The relationship between EM and CM is complex. Population-based studies have shown that compared with EM, CM is associated with greater migraine-related disability (Bigal et al., 2003), reduced quality of life, and increased medical and psychiatric comorbidities including depression (Bigal et al., 2003). Previous studies (Yong et al., 2012; Buse et al., 2013) have shown that psychiatric comorbidities, particularly depression and anxiety disorders, might be risk factors for transforming an acute migraine condition into the chronic form. This transformation can increase migraine-related disability and diminish treatment outcomes. Because there are no objective assessments for migraine, diagnosis is typically based on clinical history and the exclusion of other headache disorders. Therefore, early diagnosis and treatment of psychiatric comorbidity in migraine is very important.

Facial expression is considered a fundamental aspect of human social and emotional behavior (Gur et al., 2002). Recognition of facial expressions is very important for human communication and social cognition (Streit et al., 2003). Brain regions such as the amygdala, anterior cingulate cortex (ACC), and prefrontal cortex (PFC) are reported to play roles in processing facially expressed emotion (Adolphs, 2002; Gur et al., 2002; Brazdil et al., 2009). Previous reports (Eimer and Holmes, 2007; Burstein and Jakubowski, 2009) have shown that in addition to activation of individual brain regions, recognition of facial expression also involves interaction among several brain regions. Previous functional magnetic resonance imaging (fMRI) studies (Schwedt et al., 2015; Wang et al., 2017) have shown that people with migraines are sensitive to visual stimuli and exhibited abnormal brain activity in response to pictures of negative emotion.

Neuronal oscillatory activity represents a basic feature of the human brain, and gamma-band oscillations (30–100 Hz) in particular have been associated with distinct cognitive and sensory functions (Fries, 2009). Indeed, magnetoencephalography (MEG) revealed abnormal gamma-band oscillation in several brain regions of headache-free patients with migraine (Li et al., 2016). The responses of gamma band oscillations to emotional facial expressions have also received attention in recent years (Liu et al., 2002; Luo et al., 2007; Huo et al., 2011). Gamma band oscillations have been suggested to represent one mechanism through which sensory information is selected for processing (Basar, 2013). Thus, gamma oscillations react not only to attributes of physical stimuli, but also to the subjectively weighted percept of sensory events. Additionally, because of the short range of excitatory and inhibitory interactions elicited by incoming sensory input (Huo et al., 2011), gamma band oscillations can precisely localize brain areas involved in facial expression processing (Luo et al., 2007). The responses of gamma band oscillations are often considered to index local sensory processes within a cortical region. Therefore, gamma band oscillation offers an adequate tool for studying cortical activation patterns as different facial expressions are processed.

Magnetoencephalography can non-invasively measure neuronal activity with excellent temporal and spatial resolution, which makes it superior to scalp electroencephalography (EEG) for the spatiotemporal localization of brain processes involved in processing emotional facial expressions. Previous MEG studies (Liu et al., 2002; Xu et al., 2005) showed that facial expression processing in humans proceeds through two stages: an initial stage for face categorization and a later stage for identification of the individual face. The initial stage rapidly and automatically discriminates fearful faces from the other expressions, as evidenced by differential latencies for event-related fields (ERFs) occurring at the occipital cortex with a latency around 100 ms [M100; P1 for event-related potentials (ERPs)]. The later stage distinguishes emotional faces from neutral faces at occipito-temporal sites, peaking at about 170 ms (M170; N170 for ERPs).

The objective of the present study was to analyze how gamma-band oscillations around 100 ms (M100) and 170 ms (M170) discriminate facial expressions in people with migraine. To our knowledge, this is the first MEG study to focus on gamma-band activation elicited by emotional stimulation in interictal migraineurs using Morlet wavelet and beamformers. Building on previous reports that patients with migraine have aberrant brain responses to emotional pictures, and that gamma-band activity plays important roles in brain function, we hypothesized that patients with migraine would have aberrant gamma-band activity in response to pictures of facial emotions.

## Materials and Methods

We enrolled 70 participants, including 35 controls and 35 outpatients with migraine (15 CM and 20 EM) who were consecutively evaluated at the Nanjing Brain Hospital. All patients were diagnosed by two expert neurologists and met the International Classification of Headache Disorders, third edition, beta vision (Headache Classification Committee of the International Headache, 2013). Patients with a history of a systemic disease or any other neurological disease were excluded. Patients had not taken any medication for at least 3 days (72 h) before the MEG recording. The control participants were healthy volunteers without personal or familial history of migraine who were recruited from the community and matched for gender and age.

All participants presented normal physical and neurological examinations. The Medical Ethics Committee of the hospital approved the study protocol and each participant provided written informed consent before participation.

### Clinical and Neuropsychiatric Evaluation

All patient information was collected by neurologists and a neuropsychiatrist through a clinical interview. Clinical features for patients with migraine included the onset age, headache locus and nature, accompanying symptoms, typical duration, and frequency of headache attacks within the last year. For neuropsychiatric evaluation, symptoms of anxiety and depression were assessed in all participants using the 14-item Hamilton Anxiety Rating Scale (HAM-A) and 17-item Hamilton Depression Rating Scale (HAM-D).

### Stimuli

We used 180 gray-scale photographs of emotionally expressive faces (positive/happy, negative/fearful, and neutral) from the NimStim Set of Facial Expressions (Tottenham et al., 2009) and the Montreal Set of Facial Displays of Emotion (Beaupre, 2005) as stimuli. Mean arousal levels for both types of emotional faces were significantly higher than for neutral faces (5.70, 5.28, and 3.50 for negative, positive, and neutral faces, respectively, *F* = 177.61, *p* < 0.001). In addition to the faces, we also randomly inserted fixation-cross targets into 18 trials to ensured that participants paid attention to each image (see below for task details). Trials with fixations were not included in the MEG analysis. Emotional stimuli were back-projected onto the center of the screen through a system using BrainX software (Huo et al., 2011; Xiang et al., 2013), a video-projector placed outside of the room, and two mirrors inside the MEG room. The photographs were randomly display for 500 ms followed by 2000–2500 ms blank inter-trial intervals. The background color was set to black.

### MEG Data Acquisition

The MEG signals were recorded in a magnetically shielded room using a whole-head CTF 275-Channel MEG system (VSM MedTech Systems, Inc., Coquitlam, BC, Canada) in the MEG Center at Nanjing Brain Hospital. Patients were required to be headache-free for at least 24 h before sampling; patients who exhibited migraine symptoms during testing or during the subsequent 24 h were eliminated. Before data acquisition began, all participants were asked to remove all metal objects from their body. Three small coils were attached to the nasion and left and right pre-auricular points of the participants to check head position during MEG recordings. After that, participants comfortably lay supine during the experiment. Participants were instructed to focus their gaze on the screen (located 80 cm from there face). As they viewed the faces, they were asked to press a button with their right hand when they saw a fixation cross in the image. MEG was digitized at a sample rate of 6000 Hz with a noise cancelation of third-order gradients. MEG data were averaged from different trials and the averaged MEG data were processed with DC offset and then filtered with a 30–100-Hz band-pass filter to identify time-domain magnetic responses. Participants were asked to remain still throughout the experiment. Head position was measured at the beginning and end of the experiment. The maximum tolerated head movement was 5 mm in all sessions.

### Magnetic Resonance Imaging Scan

Anatomical image data were obtained for all participants using a 1.5T MRI scanner (Singa, GE, United States). Three fiduciary markers were placed at locations identical the positions of the three coils used in the MEG recordings. These landmarks ensured an accurate co-registration of MEG and MRI data. Subsequently, all anatomical landmarks digitized in the MEG study were made identifiable in the MRI.

### Waveform Analysis

For preliminary analysis of the time-domain waveform, MEG data were averaged to identify the evoked magnetic fields. A band-pass filter of 30–100 Hz was used for waveform analysis. The latencies and the peak amplitudes of the averaged evoked magnetic fields (deflections) M100 and M170 were automatically computed.

### Time–Frequency Analysis

Magnetoencephalography data were transformed from time-domain (waveform) to frequency-domain (spectrogram) with continuous wavelet transform (Kotecha et al., 2009; Huo et al., 2011). The Morlet continuous wavelet algorithm is defined as:

$$G(t)=C_{\sigma }\pi^{-\frac{1}{4}}e^{-\frac{1}{2}t^{2}}\left(e^{i\sigma t}-K_{\sigma }\right)$$

In the formula, *t*indicates time. K_σ_ Represents the admissibility and C_σ_ represents a normalized constant. σ Represents the standard deviation of the Gaussian curve in the time domain. Because frequency-temporal resolution changes with the value of σ, the wavelet is sensitive to frequency at low-frequencies while sensitive to time at high-frequencies. σ Was set to 6 for the 30–100 Hz frequency range used in the present study. Our study focused on the spectral power changes for two time windows (80–140 and 140–200 ms) following the presentation of affective images. These periods included the M100 and M170 components that were obtained from the time-domain waveforms in the previous analysis. To measure magnetic spectral power evoked by affective facial pictures, real-time spectrograms from 60 trails of fearful, happy, and neutral facial expressions were separately computed. The strength of the spectral power is color coded. The spectral power for all the MEG-measuring sensors was then analyzed with spectral contour maps for visualizing the spatial distribution of visually-elicited brain activity.

### Source Level Analysis

Based on previous studies (Xiang and Xiao, 2009; Xiang et al., 2013), neuromagnetic sources were estimated with wavelet-based beamformer using the following equation:

$$W_{\theta }=\frac{C^{-1}B_{\theta }}{B_{\theta }^{T}C^{-1}B_{\theta }},$$

Where C is the covariance matrix of the MEG data, and B is the forward solution for a unit current source at location θ. For beamforming analysis, multiple local spheres were fitted to each participant’s head model. A customer-designed program, MEG Processor, was used to compute and visualize magnetic sources (Kotecha et al., 2009; Xiang and Xiao, 2009). Time windows and frequency ranges for source estimation were 80–140 and 140–200 ms for signals in the 30–100-Hz frequency range. The locations of these activity sources were estimated and projected onto the structural images of the brain MRI, creating a magnetic source image (MSI) that displays the activated brain regions. We used sliding windows in the source estimation to obtain the dynamic spatiotemporal activity in the brain.

### Statistical Analysis

Data were analyzed using SPSS (IBM SPSS for Windows, version 20.0, IBM, Corp., Armonk, NY, United States). All results are expressed as mean ± standard deviation (SD). Comparisons between groups were performed with one-way analysis of variance and covariance (while controlled for age and sex), respectively, and LSD test was used for *post hoc* analysis. A Kruskal–Wallis test was conducted to evaluate the differences in the HAM-A and HAM-D ratings across the three groups, and the pair wise comparisons were conducted using the Mann–Whitney *U*-test. A chi-squared test was used to analyze gender and the locations of brain activity elicited by negative facial images across the study groups. All correlations were estimated using Spearman correlation coefficients. No multiple comparisons were performed in the statistical tests. The threshold for statistical significance was set at *p* < 0.05.

## Results

### Clinical Characteristics

Among 35 patients (25 women, 10 men) who met the inclusion criteria for the study, 20 had EM (age: 29.3 ± 8.5) and 15 had CM (age: 37.6 ± 10.5 years). 35 members of the control group (22 women, 13 men, age: 28.9 ± 7.6 years) were recruited randomly. Disease duration and duration of migraine attacks did not differ significantly between EM and CM groups (*p* > 0.05). Headache frequency was greater in the CM group than in the EM group (*p* < 0.01). HAM-A and HAM-D scores were significantly higher in patients (both EM and CM) than in healthy controls, but did not differ between the two patient groups. More detailed headache profiles and neuropsychological evaluations are summarized in **Table 1**.

**Table 1:** Clinical features and neuropsychological evaluation of patients.

Parameter	EM	CM	Control	*t*-value	*p*-value
Women/men	14/6	11/4	22/13	x^2^ = 3.29	<0.05
Age (years)	29.3 ± 8.5	37.6 ± 10.5	28.9 ± 7.6	*F* = 4.504	>0.05
Disease duration (years)	11.8 ± 7.1	12.9 ± 8.6		0.357	>0.05
Frequency (days/month)	3.4 ± 3.1	20.0 ± 15.3		-5.3	<0.001
Duration of migraine attack (h)	10.6 ± 9.3	16.7 ± 15.1		1.177	>0.05
**Accompanied symptoms with attack: % (N)**					
Phonophobia	17	13			
Photophobia	18	10			
Nausea/vomiting	14	10			
Drowsiness	5	4			
Weariness	3	2			
Sweating	0	1			
Diarrhea	0	1			
Sensitivity to smells	7	3			
Locus of headache: % (N)					
Bilateral	9	3			
Unilateral	16	12			
**Medication for treatment before MEG tests**					
Ibuprofen	20	15			
Triptans	9	3			
Percocet	15	5			
HAM-A rating	7.8 ± 2.6^##^	9.6 ± 3.6^∗∗^	1.5 ± 1.2	x^2^ = 52.324	<0.001
HAM-D rating	8.3 ± 2.4^##^	10.6 ± 3.0^∗∗^	1.5 ± 1.7	x^2^ = 52.533	<0.001


*^∗∗^*p* < 0.01 in comparison between CM group and control group, ^##^*p* < 0.001 in comparison between EM group and control group.*

### MEG Waveforms

**FIGURE 1:**
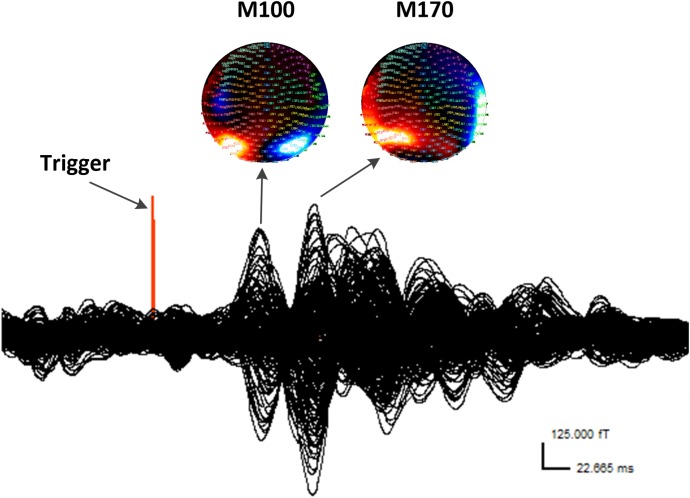
Waveform and contour maps for gamma oscillations following presentation of emotional stimuli. M100 and M170 are identifiable in the magnetic waveforms evoked by the stimulation. All magnetic field magnitude values are presented in femtotesla (fT). Red indicates incoming magnetic fields; blue indicates outgoing magnetic fields. Each small circle represents one sensor.

According to the MEG waveforms, the M100 and M170 ERFs were consistently identified in all participants (**Figure 1**). The amplitude of M170 component was significantly lower in the CM group than in the control group in response to negative emotional stimuli (*p* = 0.001), but not for positive and neutral emotional stimuli (**Table 2**). Additionally, no significant differences were found across groups or stimulus type with respect to latency (M100 or M170) or M100 amplitude.

**Table 2:** The main dependent variables analyzed in the MEG spectrograms for study groups.

Measure variables	Emotional stimuli	EM	CM	Control	*F*	*p*
Latency M100 (ms)	Fear	101.8 ± 12.3	103.3 ± 11.1	103.0 ± 11.9	0.23	0.799
	Happy	112.8 ± 22.3	106.6 ± 13.3	108.1 ± 17.0	0.89	0.414
	Neutral	109.4 ± 13.2	110.7 ± 17.2	106.4 ± 9.8	0.59	0.557
Latency M170 (ms)	Fear	157.8 ± 17.8	149.8 ± 12.4	154.4 ± 14.3	0.44	0.645
	Happy	157.0 ± 20.7	147.3 ± 15.2	160.0 ± 18.2	2.54	0.087
	Neutral	156.1 ± 20.0	155.2 ± 18.5	167.6 ± 20.8	2.70	0.075
Amplitude M100 (fT)	Fear	95.8 ± 19.8	94.4 ± 31.0	98.1 ± 26.4	0.27	0.761
	Happy	89.4 ± 28.3	81.1 ± 20.8	88.1 ± 25.0	0.64	0.532
	Neutral	89.2 ± 21.1	89.7 ± 18.5	96.3 ± 19.5	0.98	0.379
Amplitude M170 (fT)	Fear	88.3 ± 15.1	70.1 ± 9.8**	97.0 ± 27.0	8.57	**0.001**
	Happy	75.0 ± 13.2	73.2 ± 20.7	83.5 ± 21.0	5.51	0.062
	Neutral	82.6 ± 14.8	73.5 ± 22.7	88.5 ± 22.2	1.91	0.157
Spectral power value M100 (fT/Hz)	Fear	0.447 ± 0.017#	0.412 ± 0.043**	0.495 ± 0.018	3.89	**0.025**
	Happy	0.453 ± 0.016	0.476 ± 0.027	0.508 ± 0.020	1.40	0.255
	Neutral	0.473 ± 0.026	0.507 ± 0.031	0.527 ± 0.019	1.63	0.205
Spectral power value M170 (fT/Hz)	Fear	0.452 ± 0.017#	0.410 ± 0.038**	0.515 ± 0.019	5.79	**0.005**
	Happy	0.483 ± 0.023	0.540 ± 0.035	0.535 ± 0.021	1.63	0.204
	Neutral	0.429 ± 0.019	0.472 ± 0.020	0.474 ± 0.017	1.48	0.234


*^∗^*p* < 0.05 in comparison between CM group and control group, ^∗∗^*p* < 0.01 in comparison between CM group and control group, ^#^*p* < 0.05 in comparison between EM group and control group, no significant difference between EM group and CM group. Bold values represent p values still significant after post hoc analysis using the LSD test.*

### Time–Frequency Analysis

**FIGURE 2:**
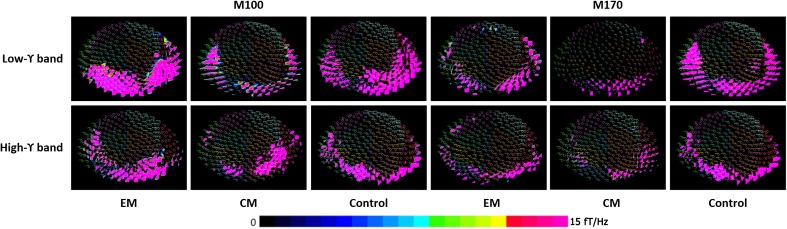
Spectral contour map of the gamma band at M100 and M170 time windows following fearful-face stimuli. The contour maps (rows 1 to 3) show the distribution of spectrograms and the active channels for the gamma-frequency band of each group. Each small circle represents one channel. The strength of spectral power is color coded. The color bar on the bottom right indicates the scale. The M100 component was primarily distributed around bilateral occipital sensors, and the M170 component was primarily localized to the bilateral occipito-parieto-temporal sensors.

Spectral contour maps showed that gamma oscillations over the time windows that included the M100 and M170 components were similar among the three groups. Gamma-band oscillations during M100 time window were predominantly observed in the bilateral occipital sensors, while those during the M170 window were located in the bilateral occipito-parieto-temporal sensors. Thus, the scalp distribution during the M100 response was slightly posterior to that of the M170 response (**Figure 2**).

**FIGURE 3:**
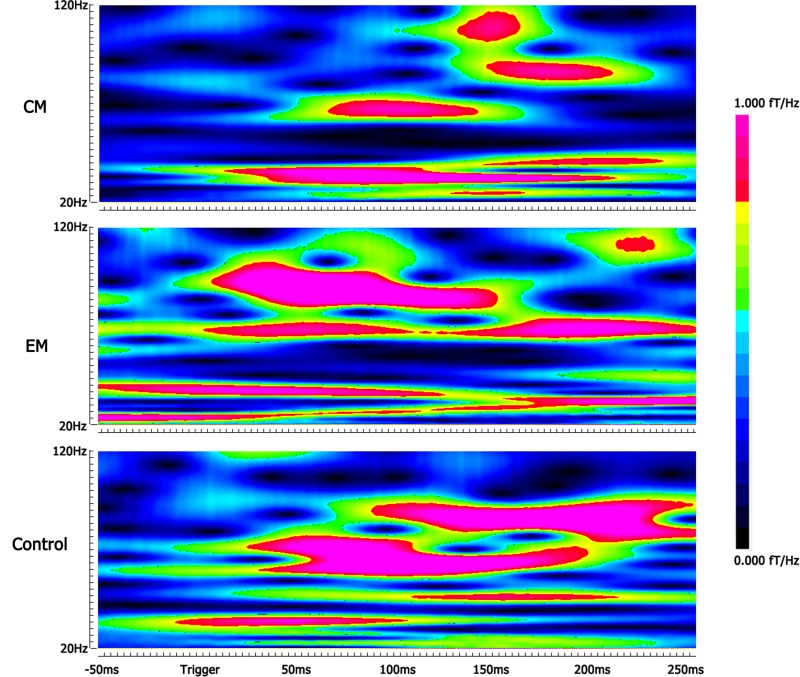
Spectrograms of gamma oscillation following the negative emotional stimuli. The real-time spectrograms show brain activity in the gamma-frequency range following the negative emotional stimuli. The color bar shows the range of spectral power values. Compared with the control group, the average spectral power was significantly lower in the EM group at M170 and in the CM group at M100 and M170.

Compared with the control group, the average spectral power was significantly lower in the EM group and CM group at M100 and M170 (*p* < 0.05, *p* < 0.05, *p* < 0.01, and *p* < 0.01) for negative emotional stimuli, but not for positive or neutral emotional stimuli. We found no significant difference in average spectral power between EM and CM groups (**Table 2** and **Figure 3**).

### Source Analysis

**FIGURE 4:**
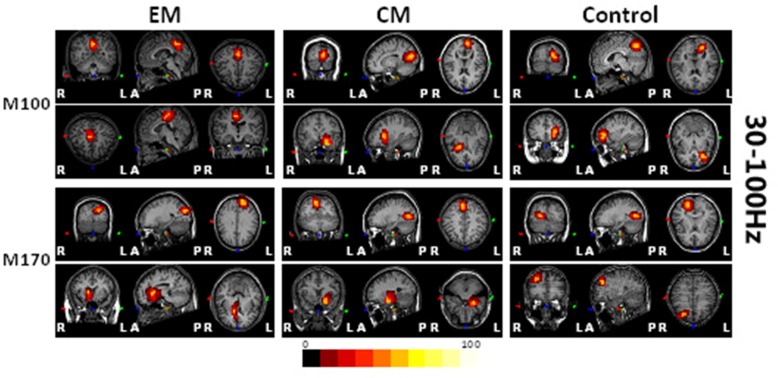
Source locations of brain activity elicited by negative emotional stimuli among the study groups. The sources of M100 and M170 were in the occipital cortex (row 1, columns 1–3) and the parietal-temporal-occipital (PTO) regions (row 3, columns 1–3), respectively for migraine and control groups. The parietal cortex (row 2, column 1) and the amygdala (row 4, column 1) were primarily activated in the EM group, the amygdala (rows 2 and 4, column 2) was primarily activated in those with migraine, while the prefrontal cortex (PFC) (rows 2 and 4, column 3) was activated in controls. The red and yellow areas indicate regions of neuromagnetic activation (or synchronized neuronal firing). The color bar indicates the strength of activation. *L*, left; *R*, right; *A*, anterior; *P*, posterior.

**FIGURE 5:**
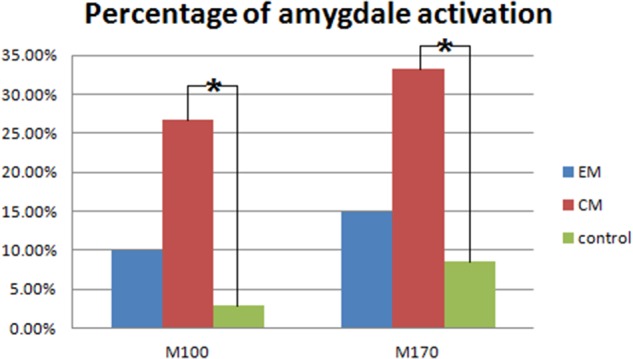
Percentage of amygdala activation. The CM group exhibited a significantly higher percentage of amygdala activation than the control group in both M100 and M170. ^∗^*p* < 0.05.

Magnetic sources for the gamma oscillations elicited by negative emotional stimuli were estimated for all patients and control participants. Source estimation was for the 30–100 Hz frequency band during M100 and M170 time windows. For the EM patient group, sources were primarily localized to the occipital cortex and the parietal cortex in M100 and to parietal-temporal-occipital area (PTO), the amygdala and the cingulate cortex in M170. For the CM patient group, they were localized to the occipital cortex and amygdala in M100 and to PTO and amygdala in M170, while for healthy controls, they were primarily localized to the occipital cortex and PFC in M100, and to PTO and PFC in M170 (**Figure 4**). The source analysis showed that after observing negative emotional stimuli, amygdala activation comprised a greater percent of the response in the CM group than in the control group (*p* = 0.002) for both M100 and M170 (see and **Table 3** and **Figure 5**). In contrast, levels of neuromagnetic activation did not significantly differ between EM and CM groups.

**Table 3:** Numbers of activated source locations in gamma oscillation.

	M100				M170		
			
Brain regions	EM	CM	Control	Brain regions	EM	CM	Control
Occipital cortex	9	6	16	Parietal-temporal-occipital regions	9	6	14
Parietal cortex	3	1	5				
Amygdala	2	4^∗^	1	Amygdala	3	5^∗^	3
Cingulate cortex	2	1	1	Cingulate cortex	3	1	3
Prefrontal cortex	2	1	8	Prefrontal cortex	2	1	9
Temporal cortex	1	1	2	Temporal cortex	2	1	3
Thalamus	1	1	2	Thalamus	1	1	3


*^∗^*p* < 0.05 in comparison between CM group and control group.*

### Clinical Correlates

Correlation analysis revealed that after the CM group viewed fearful face stimuli, the averaged gamma-band spectral power for the M100 and M170 components was significantly and negatively correlated with the HAM-D ratings (M100: *r* = -0.556, *p* = 0.031; M170: *r* = -0.749, *p* = 0.001), and attack frequency (M100: *r* = -0.351, *p* = 0.039; M170: *r* = -0.417, *p* = 0.013). In contrast, no such significant correlations were observed for the EM or control groups.

## Discussion

The present study investigated features of gamma oscillatory responses to emotional facial expression in people with migraine at times when they were not having a headache attack (interictal periods). We found that the magnitude of gamma-band oscillations in response to negative emotional stimuli were lower in patients with CMs, and MEG spectrogram analysis showed that the signal sources for these patients were different from those for without migraine. This suggests that migraineurs might not process negative emotional perceptions normally during interictal periods.

A previous study has shown that compared with other local field potential (LFP) frequency bands, visual stimulation elicits stronger gamma-band oscillations in the primate visual cortex (Berens et al., 2008). Other studies have shown that gamma-band oscillation is more sensitive to emotional stimuli than to non-emotional stimuli. Thus, gamma oscillations provide a powerful tool for understanding the brain mechanisms underlying human emotional processing (Kotecha et al., 2009; Erk et al., 2010). Previous researches also provided that different effective pictures or contexts may interfere with the pain perception (de Tommaso et al., 2009, 2013). In the present study, contour maps showed that gamma activity for the M100 component of the response was primarily distributed around bilateral occipital cortices, and we surmise that it reflects visual processing of negative emotional stimuli. The M170 response was localized in the PTO, which may reflect the recognition of faces and facial expressions. The MEG waveform analysis found that after presentation of negative emotional stimuli, but not after positive or neutral stimuli, M170 gamma amplitude was significantly lower in people with CM than in controls. Similarly, the spectrogram analysis showed that after the presentation of negative emotional stimuli, the averaged spectral power of gamma-band oscillations during the M100 and M170 time windows was significantly lower for people with CM than for controls. We also noted that after the presentation of negative emotional stimuli, patients with EM had significantly lower spectral power in gamma-band oscillations than controls in the both M100 and M170 time windows. In addition to occurring at the stage of face identification, these abnormalities were also observed at the stage of face categorization, which indicates that patients with migraine have abnormal gamma oscillatory responses to negative facial emotion during interictal periods. This supports the notion that migraine is associated with deficits in categorizing faces that express negative emotions, especially when the condition is chronic.

Another important finding was that levels of depression was relatively higher in migraineurs, which was consistent with the results from previous studies (Erk et al., 2010). We also found that after stimulating the CM group with fearful faces, the averaged spectral power in gamma oscillations for the M100 and M170 time windows were significantly correlated with HAM-D ranks. This finding suggests that the CM group had more deficits in processing facial expression stimuli than did the EM group. The abnormal responses to negative emotion might be an underlying mechanism through which emotional disorder comorbidities partly influence how the brain processes negative emotions in patients with migraine. Our findings support those from a previous study that suggested depression disorder is a risk factor for migraine chronification (Bigal, 2009). The present study also revealed that gamma-band spectral power was negatively correlated with attack frequency. Although the mechanisms underlying this phenomenon are currently unknown, we postulate that repeated migraine attacks result in abnormal brain activity related to cognitive processing of the negative emotion.

Compared with controls, those with CM exhibited a higher percentage of activation in the amygdala. The amygdala is an essential element of the limbic system and is activated by unpleasant images (Feinstein et al., 2011). Several studies have shown that amygdala activity is also related higher cognitive functions such as attention, consciousness, working memory, and long-term memory (Fried et al., 2001; Kiehl et al., 2005; Basso et al., 2006; Schaefer et al., 2006; Ousdal et al., 2008). A disorder of amygdala-cortical interactions is likely contribute to affective disorders and pain related decision-making deficits, and even pain-related cognitive impairment (Apkarian et al., 2004; Paulus, 2007; Seymour and Dolan, 2008; Pais-Vieira et al., 2009). A previous study (Stankewitz and May, 2011) demonstrated that the presence of interictal dysfunction of limbic-brainstem connectivity through the periaqueductal gray (PAG) is related to the frequency of migraine attacks and sensitization of the amygdala during migraine attack. More functional MRI studies showed that the abnormality of connections between amygdala and other brain regions may contribute to the pathogenesis of migraine and the process of pain chronicization (Androulakis et al., 2017; Chen et al., 2017; Santoro et al., 2018), even the connections between repetitive episodes of cortical spreading depression (CSD) and the development of migraine pain (Hadjikhani et al., 2013). The results of the present study have provided new evidence that abnormal response to negative emotional stimuli in the limbic system is a characteristic of migraine, especially CM. Because the brain regions involved in cognitive processing of facial expressions partially overlap with the decoding of pain (Yoshino et al., 2012), negative emotional stimuli might affect pain perception.

## Conclusion

In summary, here we show for the first time that migraine is associated with abnormal gamma oscillatory activation when viewing negative emotional stimuli. Additionally, we showed that these stimuli evoke more amygdala activation than normal when people have CM. Furthermore, the aberrant oscillatory activations were negatively correlated with depressive symptom severity and migraine attack frequency. These findings open a new avenue for us to investigate the cerebral mechanism underlying the comorbidity of depression and anxiety in migraine, which may help us to develop more effective treatments for migraine.

## Ethics Statement

This study was carried out in accordance with the recommendations of ‘guidelines of ethics committee of Nanjing Hospital (2015-KY041).’ The protocol was approved by ethics committee of Nanjing Hospital.

## Data Availability

The Primary data are included as **Supplementary Material**.

## Author Contributions

JS and TW conceived and designed the study. JX provided the technical supports. JF, YC, and DZ performed the experiments. TW recorded the MEG data. TW and JF wrote the paper. JF and YC edited the tables and figures. JS, JX, JZ, YC, and TJ reviewed and edited the manuscript. All authors read and approved the final manuscript.

## Conflict of Interest Statement

The authors declare that the research was conducted in the absence of any commercial or financial relationships that could be construed as a potential conflict of interest.
